# Thermal fluids with high specific heat capacity through reversible Diels-Alder reactions

**DOI:** 10.1016/j.isci.2021.103540

**Published:** 2021-12-07

**Authors:** Drew Lilley, Peiyuan Yu, Jason Ma, Anubhav Jain, Ravi Prasher

**Affiliations:** 1 Lawrence Berkeley National Laboratory, Berkeley, CA 94720, USA; 2 Department of Mechanical Engineering, University of California, Berkeley, CA 94720, USA; 3 Department of Chemistry, University of California, Berkeley, CA 94720, USA

**Keywords:** Thermal design, Energy engineering, Thermal engineering, Thermal property

## Abstract

Thermal fluids are used as heat transfer fluids and thermal energy storage media in many energy technologies ranging from solar thermal heating to battery thermal management. The heat capacity of state-of-the-art thermal fluids remains ∼50% of that of water (which suffers from a limited operation range between 0°C and 100°C), and their viscosities are typically more than one order of magnitude higher than that of water. Our results demonstrate that the heat capacity of the proposed thermochemical fluid is significantly higher than that of state-of-the-art thermal fluids over a broad temperature range and is also higher than that of water between 60°C and 90°C. The viscosity of our liquid is only 3 times higher than that of water, and the operating temperature range is between −90°C and 135°C. Furthermore, a model was developed allowing for novel design of thermochemical thermal fluids in the future with even higher heat capacity.

## Highlights

• Novel thermal fluid using dissolved reactants for enhanced heat capacity
• Exceeds the heat capacity of water between 60°C and 90°C
• Thermal window between −90°C and 135°C and viscosity only 3x that of water
• Molecular model inputs for *C*_*p*_ matches well with experimental data

## Graphical abstract

**Figure:**
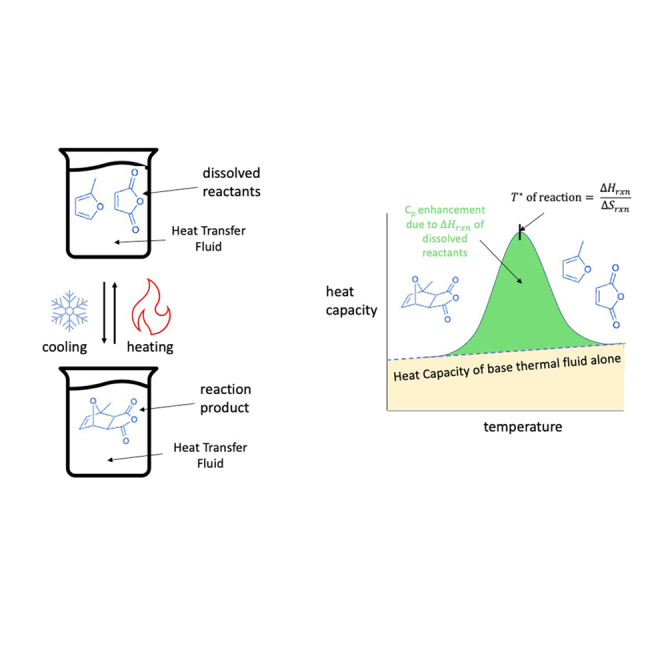

## Introduction

Approximately 90% of the world's current energy technologies involve thermal processes (Henry et al., 2020). Examples include conversion of solar energy to heat (Sharma et al., 2017), conversion of waste heat to electricity (Hung, 2001), thermal storage (Woods et al., 2021), cooling and heating of buildings (Booten et al., 2021), and thermal management of various energy devices such as batteries (Xia et al., 2017), microelectronics, and electrical transformers (Meshkatodd, 2008). For example, there is significant interest in using solar thermal processes to decarbonize industrial heating; it is expected that industrial processes requiring medium temperature (<∼150°C) heat can be economically decarbonized using solar thermal processes. For high-power microelectronics (Sohel Murshed and Nieto de Castro, 2017) and high-energy-density and fast-charging lithium ion batteries (Zeng et al., 2021), thermal management plays a very important role for reliable operation of these technologies. Other examples of thermal management include cooling of both traditional (Meshkatodd, 2008) and solid state (She et al., 2013) electrical transformers. Thermal fluids play a dominant role in all these energy technologies. Thermal fluids are used both as heat transfer fluid (HTF) and as thermal energy storage (TES) for different temperature ranges in solar thermal (Steinmann, 2020) and building applications. A good thermal fluid should possess (1) high specific heat (*C*_*p*_), (2) low freezing temperature, (3) higher boiling point (depending on the application), (4) higher thermal conductivity (k), and (5) low viscosity. Both the cooling power and thermal storage capacity of thermal fluid are proportional to *Cp*, whereas thermal and fluid resistance are dependent on thermal conductivity and viscosity, respectively. There is more freedom in designing systems with lower thermal resistance as it also depends on heat exchanger design. For example, microchannel-based heat exchangers have much smaller thermal resistance for a given fluid than larger heat exchangers (Kandlikar et al., 2013).

Among various thermal fluids used in practice and investigated in the literature, water has some of the best properties. The *C*_p_ of current thermal fluids (usually less than 2 J/g·K) has remained significantly below that of water (4.2 J/g·K). The viscosity of these thermal fluids is also much larger than that of water. Although water possesses great thermal and flow properties, the use of pure water as thermal fluid is rather limited because of its high freezing point (0°C) and low boiling point (100°C). Therefore, water is typically mixed with antifreeze liquids such as ethylene or propylene glycols, which significantly degrades its thermal properties. From a molecular point of view, water has very high *C*_*p*_ due to hydrogen bonds (10–30 kJ/mol), whereas other thermal fluids such a mineral oil have low *C*_*p*_ due to weak van der Waals bonds (<5 kJ/mol). Covalent bonds typically have bond strengths of hundreds of kJ mol^−1^. This makes thermochemical based on the formation and breaking of covalent bonds in the liquid phase very attractive as it can potentially lead to higher effective *C*_*p*_.

## Results

### Use of Diels-Alder thermochemical reaction for thermal fluids

In the 1980s, the idea of using reversible liquid-phase chemical reactions to store heat was proposed and some initial efforts were attempted (Wentworth and Chen, 1976; Lenz and Hegedus, 1979; Lenz et al., 1982; Sparks and Poling, 1983). Using calorimetry, Sparks and Poling (1983) measured the heat of reaction and equilibrium constant for the Diels-Alder reaction between maleic anhydride and dilute 2-methyl furan. Based on measured heat of reaction and equilibrium constant and an equilibrium theoretical model they proposed that a hypothetical reaction mixture at a high concentration (7 mol/L) could achieve an apparent *C*_*P*_ of 7.37 J/cm^3^·K, 76% higher than that of water. However, owing to the lack of theoretical/first principle calculations and experimental tools the potential of Diels-Alder thermal fluids was never realized and many technologically and scientifically relevant questions were never answered. Those are: (1) Direct measurement of *C*_*P*_ for Diels-Alder reactions as a function of temperature is still missing. (2) Since no measurement of *C*_*p*_ has ever been made, the impact of cycling and reversibility on the performance is not known, both of which are critical from a technological point of view. (3) Owing to the lack of experimental data, it is not clear if the equilibrium model holds or if kinetics become important. (4) Viscosity of these liquids was also not reported, which is important from the technological point of view to understand the pressure drop and pumping requirements.

The concept has since been largely overlooked by the research community and little progress has been made in this area. We recently proposed theoretically that the specific heat of liquids can be greatly enhanced by reversible chemical reactions (Yu et al., 2019) by combining density functional theory (DFT) calculations with an equilibrium thermal model. More recently, we used DFT calculations to virtually screen for thermally reversible Diels-Alder reactions that could take place in water and identified several candidate reactions for the potential applications in HTF and TES (Spotte-Smith et al., 2020).

Here, we present experimental results for the enhancement of specific heat capacity of liquids enabled by reversible Diels-Alder reactions in an organic solvent. To the best of our knowledge, this is the first direct experimental validation of such concept and offers proof-of-principle evidence for the future development of high-specific-heat storage and transfer liquids based on thermally reversible chemical reactions. We also conducted cycling experiments and viscosity measurements to answer the questions raised above. We report the results of preparing several concentrations of the 2-methylfuran and maleic anhydride in a solution of dimethylformamide (DMF) and performing calorimetry tests. We have also modified the macroscale *C*_*p*_ model based on the equilibrium model proposed by Sparks and Poling (1983) and further developed by us (Yu et al., 2019) to include chemical kinetics as the equilibrium model does not match in trend and magnitude with the experimental data. Furthermore, the macroscale *Cp* model including chemical kinetics is combined with first principles calculations using DFT for chemical parameters such as transition state enthalpy and entropy to validate the experimental results. Finally, we conclude the paper with a discussion on the future outlook and potential research directions.

**Figure 1:**
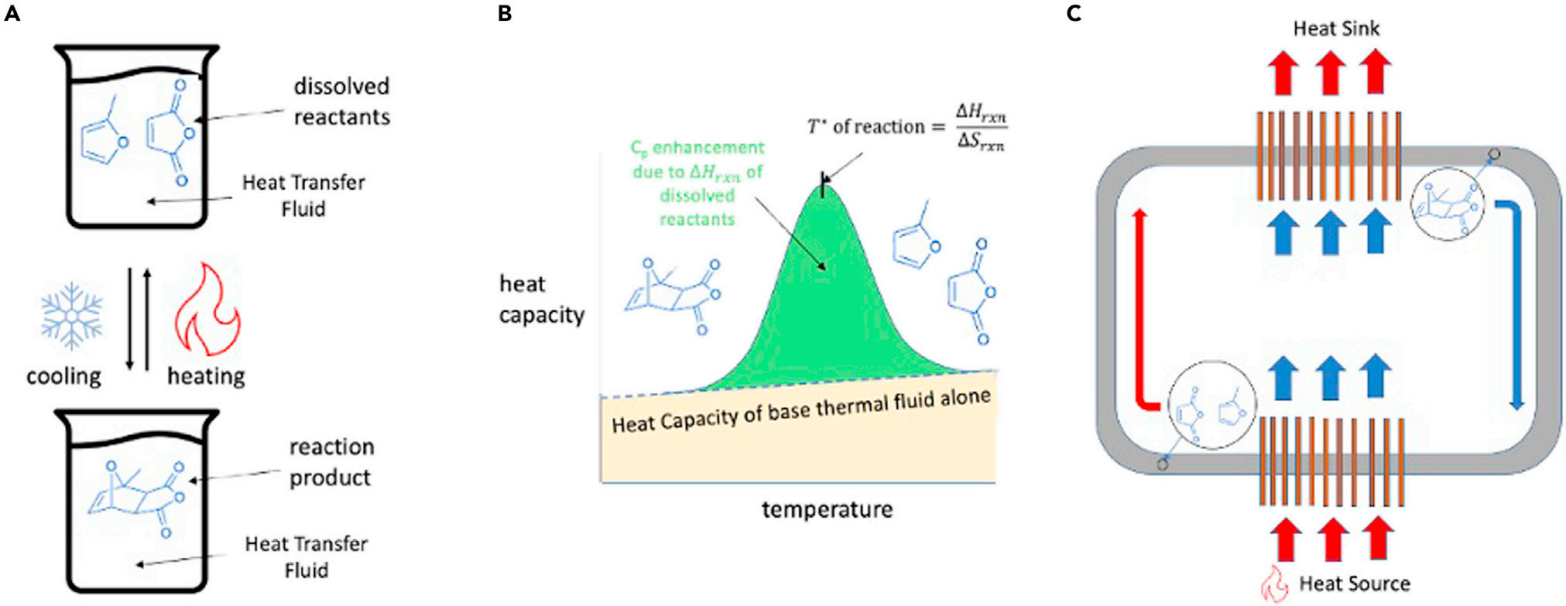
The heat capacity of a heat transfer fluid is enhanced by adding reactants that absorb energy when heated and release energy when cooled (A) shows the physical mechanism of the enhancement in which the reactants break weak covalent bonds to form a rigid ring and (B) depicts the thermodynamic effect on the heat capacity where the transition enthalpy is distributed over a large temperature range as a result of equilibrium between the reactants and products. (C) shows how the heat transfer fluid behaves at the system level, absorbing extra energy from the heat source by breaking the ring structure of the reaction product and subsequently transferring extra energy to the heat sink by re-forming the ring structure.

The basic concept for using reversible Diels-Alder reactions to enhance the specific heat of liquids is illustrated in Figure 1. Two or more reactants are dissolved in a base solution where they form some equilibrium combination of reactants and products. As the mixture is heated, excess enthalpy is required to split the product molecule into the reactants; when the mixture is cooled, this stored reaction enthalpy is released as the forward Diels-Alder reaction occurs to produce more product. Diels-Alder reactions involve a simultaneous π to σ covalent bond transition accompanying a ring formation. This process involves the breaking of two weak covalent bonds (π bond) to form two strong covalent bonds (σ bond), which result in a high Δ*H*_rxn_ as compared with that of hydrogen bonds or van der Waals interactions in conventional thermal fluids, manifesting as high heat capacities. The formation of a rigid ring from conformationally flexible structures combined with the reduction in molecularity greatly reduces the molecular degrees of freedom. This leads to a high Δ*S*_*rxn*_ within the liquid phase. The high Δ*S*_rxn_ is critical because it ensures that the turning temperature of the reaction, T∗, is not too high and remains within the liquid temperature range of the mixture. Thus, although many types of liquid phase chemical reactions could in theory achieve high ΔH_rxn_ alone, Diels-Alder reactions are ideal for this concept because they also have high Δ*S*_rxn_.

### Experimental results of Diels-Alder enhanced thermal fluid

**Figure 2:**
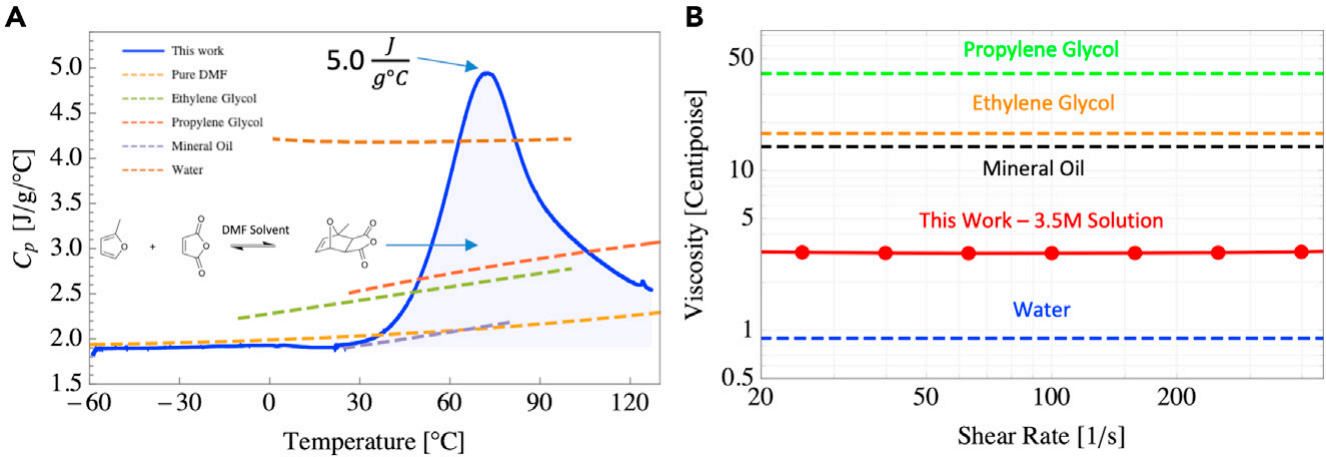
Effective heat capacity and viscosity of the investigated Diels-Alder fluids (A) Effective heat capacity of 3.5 M maleic anhydride and 2-methylfuran in DMF (solid blue) compared with the heat capacities of common thermal fluids (water, ethylene glycol, propylene glycol, mineral oil, and pure DMF). The blue shaded region indicates the enhancement provided by the chemical reaction taking place between the maleic anhydride and 2-methylfuran. (B) Viscosity of the 3.5 M mixture over shear rates 20–600 1/s showing a Newtonian response. We include water, mineral oil, ethylene glycol, and propylene glycol data for comparison.

**Figure 3:**
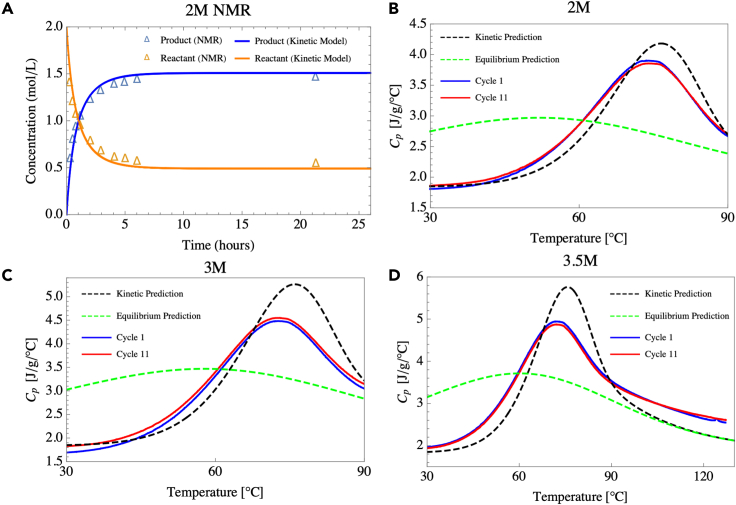
Cycling data and comparisions with DFT calculations (A) NMR data (triangles) showing the concentration versus time of the maleic anhydride and 2-methylfuran isothermal reaction at 26°C. The solid lines indicate the predictions given by Equation (2) using inputs determined from density functional theory calculations. (B–D) DSC measurements of 2, 3, and 3.5 M mixtures. The blue solid line indicates the first measurement, and the red solid line shows the performance after 10 heating and cooling cycles. The black dashed line shows the predicted heat capacity using Equation (1) (see STAR Methods) and the green dashed line shows the predicted equilibrium heat capacity from previous work (Yu et al., 2019). The thermodynamic inputs were determined from density functional theory calculations.

The effective heat capacity of 2, 3, and 3.5 M mixtures of maleic anhydride and 2-methylfuran in DMF were determined using differential scanning calorimetry (see STAR Methods). We begin by discussing the properties of the 3.5 M mixture as plotted in Figure 2A. The region of heat capacity enhancement over that of DMF alone is concentrated between 30°C and 120°C, which is sufficiently large for many heat transfer applications. The heat capacity enhancement region is represented by the blue shaded region in Figure 2A. The 3.5 M solution has an enthalpy change of ∼345 J/g from 20°C to 135°C, which is about 99 J/g greater than that of pure DMF (∼246 J/g) and represents a total enthalpy enhancement of approximately 40% due to the presence of the reactive species. The heat capacities of the 3.5 M mixture, pure DMF, ethylene glycol, propylene glycol, mineral oil, and water are also compared in Figure 2A. The heat capacity of the 3.5 M mixture exceeds that of even water (4.18 J/gC) from ∼60°C to 90°C, peaking at $∼5.0\frac{J}{g{}^{\circ}C}$, which is approximately 20% higher than that of water. Figure 2A shows that the heat capacity of the 3.5 M mixture is substantially higher than the heat capacity of propylene glycol and ethylene glycol from ∼45°C to 100°C, demonstrating the large improvement in the energy transfer and storage capacity over traditional heat transfer fluids. We emphasize that we do not expect that this mixture will replace water particularly in the temperature range for which water is well suited. The goal of comparison with water, which has excellent thermophysical properties and widespread use, was to help show the possibilities of the reactive mixture over a base fluid. In particular, we have demonstrated through this study that it is possible to achieve both an expanded temperature range from water and high specific heat capacity, which is difficult to achieve using any single fluid, thus highlighting the usefulness of the concept in general. We also note that the thermograms in Figures 2 and 3 are sensitive to the heating rate, and the sensitivity is captured in Equations (1) and (2). The heating rate of $10\;\frac{{}^{\circ}C}{min}$ was used to generate Figures 2 and 3 and was chosen out of experimental convenience.

The viscosity of the mixtures was measured using a standard parallel plate rheometer and is plotted in Figure 2B and Equation (2). The mixtures remain approximately Newtonian for both high- and low-shear regimes, making them favorable for thermal applications ranging from thermal storage (slow shear rate) to classical applications in heat transfer fluids (high shear rate). Pure DMF exhibits a very low viscosity of 0.8 mPa-s at *T* = 25°C, roughly equal to that of pure water, making it an excellent choice as a base fluid to host the Diels-Alder reactants. The viscosity increases approximately linearly with increasing reactant concentration (see Equation 2) and at 3.5 M is equal to 3.04 *mPa*−*s* @ *T* = 25°C. This represents a significant improvement over other traditional heat transfer fluids, such as propylene glycol (40.4 *mPa*−*s*), ethylene glycol (17.1 *mPas*), 50/50 water and propylene glycol (4.5 *mPa*−*s*), and mineral oil (14.1 *mPa*−*s*), all taken at room temperature. Thus, such mixtures are attractive not only for their enhanced heat capacity but also potentially for their flow properties.

### Predictions using DFT

We note that the properties of these mixtures can be accurately predicted from first-principles calculations, making it feasible to design such mixtures using different reactive species *in silico* (Spotte-Smith et al., 2020) and also confirming mechanistic understanding of the heat capacity enhancement. For example, in Figure 3A we compare nuclear magnetic resonance (NMR) measurements of the time-dependent concentration profiles of 2 M maleic anhydride and 2-methylfuran in DMF at 26°C against predictions made using the molecular parameters in Table 1 determined from DFT calculations (see STAR Methods). The kinetic model (see Equation 2) tracks the NMR-determined concentration profiles very closely for both the rapid transience observed over short time scales (t < 10 h) and the asymptotic approach toward equilibrium thereafter. The agreement, which is obtained without any fitting parameters, demonstrates that the reactions indeed occur as would be expected based on the fundamental properties of the overall reaction.

**Table 1:** Calculated properties from density functional theory of the reaction between maleic anhydride and 2-methylfuran, which are used to model the expected heat capacity curves

	Δ*H*_*rxn*_[Kcal/mol]	Δ*S*_*rxn*_[cal/mol]	Δ*H*^‡^[Kcal/mol]	ΔS^‡^[cal/mol]
2-Methylfuran + maleic anhydride	−14.44	44.63	12.04	−35.88

Furthermore, the theoretical calculations allow prediction of the full heat capacity profile. Using again the molecular parameters in Table 1 determined from DFT, we compare in Figures 3B–3D the model predictions to experimental heat capacity data. Figures 3B–3D show that the *C*_*p*_ model based on the equilibrium model previously proposed by Sparks and Poling (1983) and further developed by us (Yu et al., 2019) grossly underrepresents the data. At infinitely slow ramp rates, the system sees a heat capacity enhancement between about 0°C and 120°C, with the peak enhancement around 50°C. As the ramp rate increases, the heat capacity enhancement occurs at higher temperatures in a more concentrated temperature range, as shown in Figure 3. Note that the equilibrium model only depends on Δ*H*_*rxn*_ and Δ*S*_*rxn*_ given in Table 1. To resolve this discrepancy, we modify the macroscopic *C*_*p*_ model to account for chemical kinetics (see STAR Methods), which depends on all four parameters given in Table 1. Given that the theoretical prediction using kinetic model uses no empirical fitting parameters, the agreement is quite good for the 2 M concentration (peak heat capacity difference of 6.9%, temperature of peak heat capacity difference of 2.2°C). However, the model shows larger deviations for the 3.5 M concentration; in particular, the experimental peak heat capacity enhancement is not as high as expected. This could potentially be due to mass transport limitations (Wang et al., 2020), and further investigation is needed to determine if the full theoretical heat capacity can be unlocked at high concentrations. Nevertheless, the excellent agreement validates the DFT determined molecular parameters and is encouraging for future DFT-based thermochemical screening efforts. Moreover, it enables the prediction of system-level heat capacity profiles by coupling the solution of the heat equation to the reaction kinetics, which depend on the temperature versus time. We note that the thermograms in Figures 2 and 3 are specific to the DSC system, where no thermal gradients exist, and the heating rate was prescribed at $10\frac{{}^{\circ}C}{min}$. In general, the thermogram will appear different depending on both the heating rate and thermal boundary conditions, as captured by Equation (1) in the STAR Methods section.

All samples were cycled 11 times, and the effective heat capacity of the first and last cycles are plotted in Figures 3B–3D. No appreciable degradation was observed over the 11 cycles, indicating a highly reversible reaction. Finally, we note that the thermal window of the Diels-Alder mixtures is greatly enhanced relative to water. The freezing point of pure DMF is −61°C. However, with a high concentration of reactants dissolved into it as in our work, it exhibits a large freezing point depression as no nucleation was observed as low as −90°C. Although the boiling point of pure DMF is *T* = 153°C, we observed boiling at *T* = 145°C; we attribute this to the 2-methylfuran boiling, given its low nominal boiling point out of solution (63°C). The effective thermal window for the Diels-Alder mixtures is then −90°C to 145°C, which is much larger than that of water and traditional water-glycol mixtures.

Overall, the mixtures exhibited enhanced heat capacity (and thereby greater stored enthalpy) over 11 cycles, low viscosity, and an extended thermal stability window as compared with typical heat transfer fluids. A summary of the properties are listed in Table 2.

**Table 2:** Comparison of energy stored and other properties of 3.5 M mixture versus water and 50/50 propylene glycol and water mixture

	This work [3.5 M]	Water	50/50 propylene glycol and water mixture
E_stored_ (J)	354.5 (20–135°C)	314.2 (20–95°C)	315.8 (20–105°C)
Viscosity @25°C (mPa-s)	3.04	0.8	4.53
C_p,avg_ (J/kg-°C)	3.08 (20–135°C)	4.19 (20–95°C)	3.70 (20–105°C)
C_p,max_ (J/kg-°C)	5.00	4.21	3.86
Melting temperature (°C)	−90	0	−34
Boiling temperature (°C)	145	100	105

### Future outlook

We have demonstrated that by dissolving a reactive species in a base solution, we can greatly enhance the effective heat capacities of thermal fluids. To further improve the performance of these thermal fluids and look toward their application as energy storage and heat transfer materials, we must seek higher-energy-density mixtures. Good agreement between the DFT model and experimental data allows for quick screening of existing molecules as well as design of new molecules for enhanced *C*_*p*_. From the theoretical model, there are six parameters that decide the heat capacity of the thermochemical fluid: (1) the base heat capacity of the solution, (2) the heat of reaction (ΔH_rxn_) and the entropy of reaction (ΔS_rxn_), (3) the transition state enthalpy (Δ*H*^‡^f) and the transition state entropy (Δ*S*^‡^f), and (4) the solubility of the reactants in the solvent. The overall energy density can be improved in several ways: (1) Using a solution with higher base heat capacity, such as using water as solvent. In a previous work (Spotte-Smith et al., 2020) where we theoretically screened potential reactants using DFT for use in an aqueous solvent, although high heat capacities were theoretically possible, the temperature range of such solutions will likely be limited by the liquid range of water. (2) Using existing molecules or designing molecules with high ΔH_rxn_ and appropriate Δ*H*^‡^f and Δ*S*^‡^f for better kinetics but retaining an appropriate turning temperature by also increasing ΔS_rxn_. In our previous computational study (Spotte-Smith et al., 2020) using DFT we screened existing molecules as well as evaluated the performance of new molecules using various functional groups such as methyl, methoxy, and formyl groups. Although our previous work did not take kinetics into consideration, it showed that the design space for Diels-Alder thermochemical thermal fluids is huge.

We have conducted experiments in a static manner to measure *C*_*p*_; however, in application settings HTFs will be flowing. The impact of advection on reaction kinetics and solubility and how it impacts effective *Cp* remains unknown. A system with a condensed phase reaction occurring during flow is a complex system both from molecular (Wang et al., 2020) and macroscopic points of view. One will have to develop coupled thermal, fluidic, and chemical macroscopic model to understand the system-level performance of these thermochemical HTFs, in contrast to traditional fluids with monatomically increasing heat capacity that can be modeled with standard finite element packages. From a molecular point of view, the reaction rate may increase due to increased diffusion of species (Wang et al., 2020). Analogous to the static model where we have combined macroscale thermodynamic and kinetic model with molecular models of basic chemical parameters, potentially a dynamic model can be developed. Some of these open questions should be explored after conducting a thorough study of these thermochemical HTF in flowing conditions.

### Limitations of the study

We have demonstrated through this study that it is possible to achieve both an expanded temperature range from water as well as high specific heat capacity, which is difficult to achieve using any single fluid, thus highlighting the usefulness of the concept in general. However, the thermograms in Figures 2 and 3 are sensitive to the heating rate, and the sensitivity is captured in Equations (1) and (2). A more sophisticated model should be developed and used to study the behavior of this mixture in more realistic systems (e.g., heat exchangers, thermal storage) where non-uniform temperature distributions are common and the heating/cooling rate varies as a function of time and/or position.

## STAR★Methods

### Key resources table

**Table:** 

REAGENT or RESOURCE	SOURCE	IDENTIFIER
**Chemicals, peptides, and recombinant proteins**		

2-methylfuran	Sigma Aldrich	M46845-100ML
Maleic Anhydride	Sigma Aldrich	M188-25G-A
*N*,*N*-Dimethylformamide	Sigma Aldrich	227056-100ML

### Resource availability

#### Lead contact

Further information and requests for resources and should be directed to and will be fulfilled by the lead contact, Ravi Prasher (rsprasher@lbl.gov).

#### Materials availability

This study did not generate new unique reagants

### Method details

#### Heat capacity measurements

The heat capacity was measured using differential scanning calorimetry (DSC) at a scan rate of $10\frac{{}^{\circ}C}{min}$ with 10*μL* samples in hermetically sealed non-reacting pans. The heat capacity measurements were calibrated using NIST reference data for DMF, sapphire, and water (Domalski and Hearing, 2021).

#### Cycling procedure

After initial mixing, each solution was equilibrated at 25°*C* for 12 hours. Once equilibrated, the solutions were cycled in a DSC between −60°*C* and 135°*C* 10 times at both a heating and cooling rate of $10\frac{{}^{\circ}C}{min}$. After 10 cycles, the solutions were again equilibrated at 25°*C* for 12 hours, and then ramped from −60°*C* to 135°*C* at $10\frac{{}^{\circ}C}{min}$.

#### DFT molecular modeling to predict transition states

Initial molecular structures of the reactants and product were constructed using Avogadro (Avogadro: an open-source molecular builder and visualization tool. Version 1.2.0. http://avogadro.cc/) and optimized using the MMFF94 force field (Halgren, 1996). These initial structures were then optimized using the ωB97X-D (Chai and Head-Gordon, 2008) density functional and the 6-31G basis set as implemented in the Q-Chem 4.4 software package using the keyword OPT. The transition state structure was constructed from the product structure by elongating the forming bonds to 2.0 Å and then optimized using the keyword TS. Subsequent vibrational frequencies were calculated using the keyword FREQ to obtain entropies and thermal corrections for enthalpy at 298.15 K. Single-point energy calculations were performed with a larger basis set 6-311++G(d,p) and with the PCM implicit solvation model (Mennucci et al., 2002) (dielectric constant ε = 38.25 for DMF (Lide, 2004)).

#### NMR

The samples for NMR analysis were prepared by dissolving 2-methylfuran (1 mmol) and maleic anhydride (1 mmol) in 500 μL of DMF-*d7*. ^1^H NMR spectra were recorded on a Bruker 300 or 400 MHz spectrometer. ^1^H chemical shifts are reported in parts per million relative residual protiated solvent as a reference (DMF in DMF-*d7*: 2.86 ppm).

#### Kinetic heat capacity model

The Eyring equation (Espenson, 1995) was used to describe the change in concentration with time in Eq. 2, and was solved using the equilibrium concentration governed by the Van't Hoff equation (Atkins and De Paula, 2006) as the initial condition. The equilibrium heat capacity as a function of reactant concentration and temperature can be related (Yu et al., 2019) to the chemical equilibrium governed by the Van't Hoff equation, which relates the equilibrium constant (*K*_eq_) to the enthalpy (Δ*H*_rxn_) and entropy (Δ*S*_rxn_) of reaction. Heat transfer fluids are typically heated and cooled very rapidly, so in a typical application the state of the chemical reaction will be far from equilibrium. To account for the kinetics, the number of bonds formed over a temperature interval multiplied by the enthalpy change associated with bond formation can be related to the chemical-based enhancement to the heat capacity of the solution at a given temperature:

(Equation 1) $$C_{p,kinetic}\left(T\right)=\frac{\text{Δ}H_{rxn}}{\rho }\frac{dc}{dT}+C_{p,\;fluid}=\frac{\text{Δ}H_{rxn}}{\rho }\frac{\left(\frac{dc}{dt}\right)}{\left(\frac{dT}{dt}\right)}+C_{p,\;fluid}=\frac{\text{Δ}H_{rxn}}{\rho }\frac{\left(\frac{dc}{dt}\right)}{\beta }+C_{p,\;fluid}$$

where *β* is the ramp rate in the DSC. In general, $\frac{dc}{dt}$ can only be decoupled from $\frac{dT}{dt}$, or *β* when the Biot number is less than 0.1, such that $Bi=\frac{hL}{k}<0.1$, where *h* is the heat transfer coefficient, *L* is the length scale, and *k* is the thermal conductivity. In our DSC experiments, *L* ≈ 1*mm*, $k\approx 1\frac{W}{mK}$, and if we assume free convection (most pessimistic case), *h* ≈ 10 so that *Bi* ≈ 10∗0.001 ≈ 0.01, so this assumption holds and $\frac{dc}{dt}$ can be decoupled from β. In Equation (1), *c* is the molar concentration which can be predicted by kinetic theory (see Equation 2), *T* is the temperature in Kelvin, $\text{Δ}H_{rxn}=H_{product}-\sum H_{reactant}\;$, *ρ* is the density of the mixture, and $C_{p,fluid\;}=\chi_{base\;fluid}C_{p,base\;fluid}+\underset{i}{\sum }\chi_{i}C_{p,i}$ where *χ* represents the volume fraction of a given species.

#### Equilibrium and kinetic thermodynamic model to predict energy storage enhancement

Upon heating, the dissolved species reversibly react to form covalent bonds. At each temperature there exists an equilibrium between the reactants and products dissolved in solution, and the change in that equilibrium with the change in temperature determines the number of reactants that form products over a given temperature interval. The change in concentration can be related to the change in heat capacity of a thermal fluid. In reality, heat transfer fluids are heated/cooled quickly so the reaction is unlikely to observe equilibria at a given temperature. More generally, then, the number of bonds formed over a temperature interval multiplied by the enthalpy change associated with bond formation can be related to the chemical-based enhancement to the heat capacity of the solution at a given temperature by Equation (1) in the main text.

To evaluate Equation (1) in STAR Methods (main manuscript), we can predict the change in concentration with time (i.e. kinetics of the reaction) using the Eyring equation:

(Equation 2) $$\frac{dc}{dt}=\frac{k_{b}T\left(t\right)}{h}e^{-\frac{\text{Δ}{\text{G}}_{\text{f}}}{RT\left(t\right)}}{\left(c_{\text{max}}-c\left(t\right)\right)}^{2}-\frac{k_{b}T\left(t\right)}{h}e^{-\frac{\text{Δ}G_{rev}}{RT\left(t\right)}}c\left(t\right)$$

Where *k*_*b*_ is the Boltzmann constant, *h* is Planck's constant, *T*(*t*) = *T*_*o*_+*βt*, Δ*G*_*f*_ = Δ*H*_*f*_−*T*Δ*S*_*f*_ and $\text{Δ}G_{rev}=\left(\text{Δ}H_{f}-\text{Δ}H_{rxn}\right)-T\left(\text{Δ}S_{f}-\text{Δ}S_{rxn}\right)$. Equation (2) is a nonlinear first order ODE. We choose the equilibrium concentration of reactants as the initial condition, which can be derived from the Van't Hoff equation:

(Equation 3) $$c\left(T_{o}\right)=\frac{1}{2}\left(2c_{\text{max }}+\frac{1}{K_{eq}\left(T_{o}\right)}\right)-\frac{1}{2}\sqrt{\left(2c_{\text{max }}+\frac{1}{K_{eq}\left(T_{o}\right)}\right)-4c_{max}^{2}}$$

*T*_*o*_ is the initial temperature at which the solution was equilibrated and *c*_*max*_ is the concentration of the reactants before the reaction has begun. Equation (2) can then be numerically solved using Equation (3) as the initial condition, the result of which can be inserted into Equation (1) of the main text to determine the heat capacity as a function of temperature for a given starting concentration of reactants, and at a prescribed heating/cooling rate.

## Data Availability

Raw data reported in this paper will be shared by the lead contact upon request. This paper does not report original code. Any additional information required to reanalyze the data reported in this paper is available from the lead contact upon request.
