# Supplementary material for: Thermal fluids with high specific heat capacity through reversible Diels-Alder reactions
Source: iScience. 2021 Dec 7;25(1):103540. doi: 10.1016/j.isci.2021.103540 (PMC8715154; doi:10.1016/j.isci.2021.103540)

iScience, Volume *25*

Supplemental information

Thermal fluids with high specific heat capacity through reversible Diels-Alder reactions

Drew Lilley, Peiyuan Yu, Jason Ma, Anubhav Jain, and Ravi Prasher

Supplementary Information

Figure S1: Extension of Figure2b -- *Viscosity of 2.0M, 3.0M, and 3.5M solutions.*

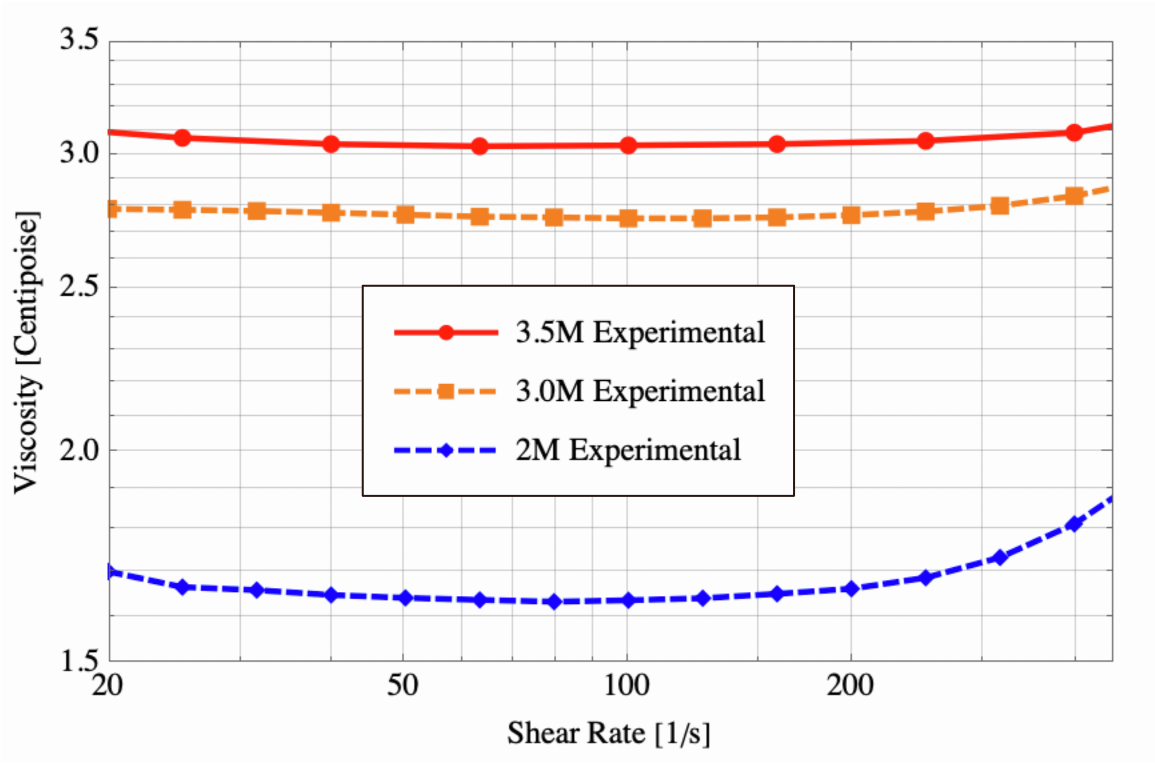

Supplement: Document S1. Figure S1 [file mmc1.pdf]
